# The sweet trap in tumors: aerobic glycolysis and potential targets for therapy

**DOI:** 10.18632/oncotarget.7676

**Published:** 2016-02-24

**Authors:** Li Yu, Xun Chen, Liantang Wang, Shangwu Chen

**Affiliations:** ^1^ Department of Pathology, The First Affiliated Hospital, Sun Yat-sen (Zhongshan) University, Guangzhou, P.R. China; ^2^ Guanghua School of Stomatology, Hospital of Stomatology, Sun Yat-sen University, Guangzhou, P.R. China; ^3^ State Key Laboratory for Biocontrol, Guangdong Key Laboratory of Pharmaceutical Functional Genes, Department of Biochemistry, School of Life Sciences, Sun Yat-sen (Zhongshan) University, Guangzhou, P.R. China

**Keywords:** aerobic glycolysis, Warburg effect, glucose metabolism, targets for the tumor therapy

## Abstract

Metabolic change is one of the hallmarks of tumor, which has recently attracted a great of attention. One of main metabolic characteristics of tumor cells is the high level of glycolysis even in the presence of oxygen, known as aerobic glycolysis or the Warburg effect. The energy production is much less in glycolysis pathway than that in tricarboxylic acid cycle. The molecular mechanism of a high glycolytic flux in tumor cells remains unclear. A large amount of intermediates derived from glycolytic pathway could meet the biosynthetic requirements of the proliferating cells. Hypoxia-induced HIF-1&alpha;, PI3K-Akt-mTOR signaling pathway, and many other factors, such as oncogene activation and tumor suppressor inactivation, drive cancer cells to favor glycolysis over mitochondrial oxidation. Several small molecules targeting glycolytic pathway exhibit promising anticancer activity both *in vitro* and *in vivo*. In this review, we will focus on the latest progress in the regulation of aerobic glycolysis and discuss the potential targets for the tumor therapy.

## INTRODUCTION

In addition to morphological change and function loss, another obvious change is cellular energy metabolism during the transition from a normal cell to cancer cell. Deregulated energy metabolism has therefore been recognized as one of the hallmarks of cancer [1]. Abnormal glucose metabolism is well known in tumor cells and characterized with aerobic glycolysis. Glycolysis is a critical catabolic process of the glucose, which breaks down one molecule of glucose to produce two pyruvates together with two ATPs and two reduced nicotinamide adenine dinucleotide (NADH) molecules. The fate of pyruvate largely depends on the supply of oxygen for the cells. Pyruvate is transformed into lactate in the absence of oxygen *via* an anaerobic glycolysis pathway. In contrast, pyruvate is oxidized to CO_2_ and H_2_O in the presence of oxygen through the oxidative phosphorylation (OXPHOS) pathway, resulting in the production of large amounts of ATP. It was found in nearly one century ago that tumor cells have a much higher rate of glucose consumption [2]. Most cancer cells produce large amounts of lactic acid regardless of the availability of oxygen [3]. This phenomenon converted glucose to lactate in the presence of oxygen is known as the Warburg effect or aerobic glycolysis [4]. This review will update the latest progress on the regulatory mechanism of the Warburg effect and discuss the potential targets in this pathway for the tumor therapy.

## PATHWAY OF GLYCOLYSIS

**Figure 1:**
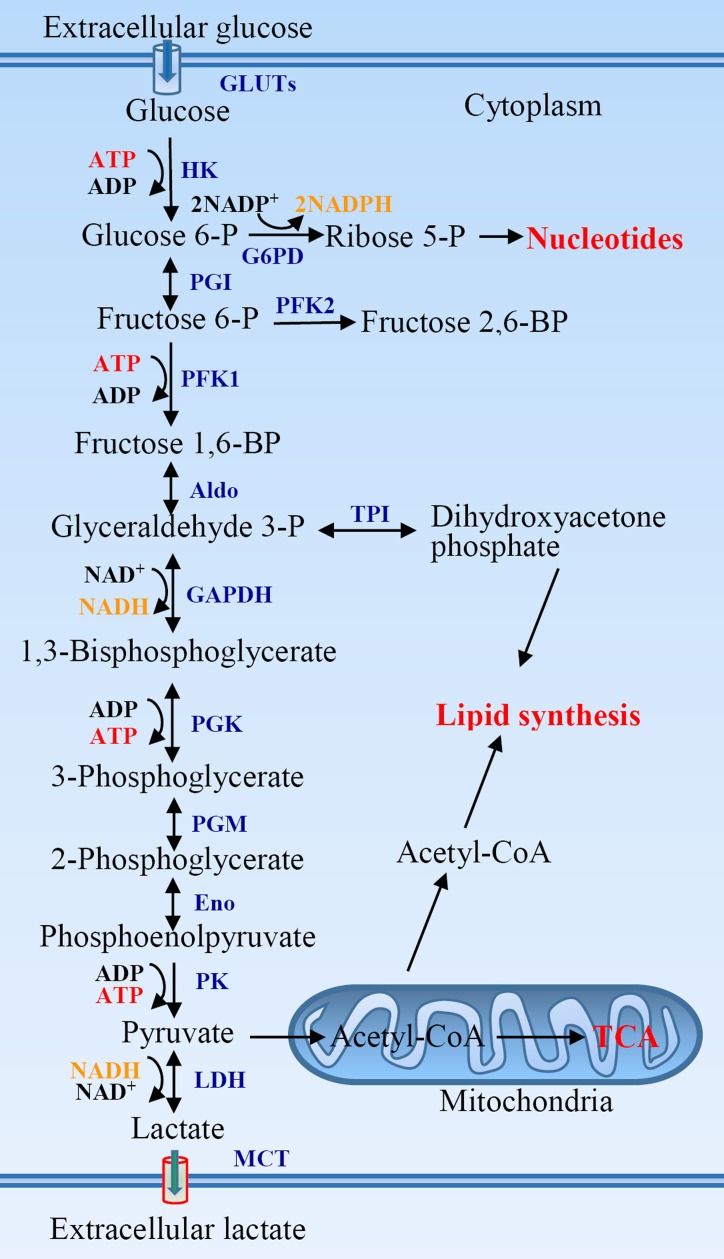
The glycolytic pathway and its association with other metabolic pathways Aldo, aldolase; Eno, enolase; G6PD, glucose-6-phosphate dehydrogenase; GAPDH, glyceraldehyde 3-phosphate dehydrogenase; GLUTs, glucose transporters; HK, hexokinase; LDH, lactate dehydrogenase; MCT, monocarboxylate transporter; PFK, phosphofructokinase; PGI, phosphoglucose isomerase; PGK, phosphoglycerate kinase; PGM, phosphoglycerate mutase; PK, pyruvate kinase; TCA, tricarboxylic acid cycle; TPI, triose phosphate isomerase.

A molecule of glucose is degraded to two molecules of three-carbon pyruvate in ten steps in glycolysis. Glycolysis pathway initiates from the phosphorylation of glucose to glucose-6-phosphate (Figure 1). Glucose-6-phosphate is then converted to fructose-6-phosphate that is further phosphorylated to form fructose-1,6-biphosphate. Two molecules of ATP are consumed in two steps of phosphorylation. Fructose-1,6-biphosphate is then broken to yield two three-carbon tautomeric molecules, dihydroxyacetone phosphate and glyceraldehyde 3-phosphate. Glyceraldehyde 3-phosphate is oxidized and phosphorylated to form 1,3-biphosphoglycerate by inorganic phosphate in the payoff phase of glycolysis. The latter is converted to 3-phosphoglycerate yielding a molecule of ATP *via* substrate-level phosphorylation. 3-phosphoglycerate is isomerized to 2-phosphoglycerate that is dehydrated to produce phosphoenolpyruvate. Phosphoenolpyruvate is subsequently converted to pyruvate yielding a molecule of ATP again. Two molecules of glyceraldehyde 3-phosphate are oxidized to two pyruvates, in which four molecules of ATP are produced *via* two steps of substrate-level phosphorylation. The net yield is two molecules of ATP per molecule of glucose oxidized because two ATPs are invested in the preparatory phase. Pyruvate is reduced to lactate under hypoxia, or oxidized to yield acetyl-coenzyme A under aerobic condition and then oxidized completely to CO_2_ *via* citric acid cycle.

## AEROBIC GLYCOLYSIS IN TUMORS

One of main metabolic characteristics of cancer cells is aerobic glycolysis, the high level of glycolysis even in the presence of oxygen. Hypoxia is supposed to be a main reason driving tumor cells to anaerobic glycolysis pathway. Hypoxia and glucose shortage occur in the inner mass of a growing tumor due to supply of blood. Glycolytic switch is actually acquired very early in carcinogenesis, even before tumors experience hypoxia [3]. It is likely that two early steps in transformation of a normal cell into a tumor cell are the change to dependence on glycolysis for ATP production and the development of tolerance to a low pH in the extracellular environment caused by release of lactate. Lung cancers and leukemic cells are growing in the presence of oxygen. Even so, they still drive glucose into the aerobic glycolysis pathway [5, 6]. Many tumors utilize aerobic glycolysis to meet their metabolic requirements even in normoxic conditions, suggesting that the Warburg effect is not solely adaptive to hypoxia. Actually, glycolysis facilitates tumor growth, and reversing the glycolytic phenotype to OXPHOS in cancer cells can promote cell death [7]. The increased take-up and consumption of glucose in tumors is observed compared with normal tissues, which has been used to identify tumors and metastatic lesions by positron emission tomography [8] and currently new developed methods [9, 10].

It seems reasonable that the metabolic switch from OXPHOS to glycolysis during hypoxia or mitochondrial dysfunction is critical for cancer cell growth [11, 12]. Mitochondrial impairment and subsequent defective OXPHOS is frequently found in cancers [13]. It is clear that mitochondrial defect in cancer cells can cause a shift in energy metabolism, in which hypoxia-inducible factor-1α (HIF-1α) plays an important role as an activator of aerobic glycolysis and lactate production. However, most tumor cells display a normal mitochondrial function including normal capacity for mitochondrial OXPHOS [14–16]. The high glycolytic activity in cancer cells does not mean a reduction in OXPHOS [17]. It was reported that ATP production is 80% oxidative and 20% glycolytic in breast cancer and glioma cells [14–16]. The contribution of aerobic glycolysis in these cells is similar to that in non-transformed cells. In contrast, mtDNA gene mutations reduce colony formation and growth rate of cancer cells and diminish tumorigenicity [18], which is largely discriminated from typical characteristics of cancer cells. Thus glycolytic switch in tumors is subjected to a complex regulation (see discussion below).

It is obvious that glycolysis yields a lower amount of ATP compared to mitochondrial OXPHOS. Why do tumor cells prefer to fuel glucose to the aerobic glycolysis pathway? Several key benefits inherent in aerobic glycolysis drive cancer cells to favor glycolysis over mitochondrial oxidation [13, 19]. First, the glycolysis leads to faster ATP production. The rate of ATP production may be 100 times faster with glycolysis than with OXPHOS [20]. The increased rate of ATP production resulting from glycolysis confers a selective growth advantage to cancer cells [21, 22]. Second, high glycolytic rates likely benefit rapid proliferating cells through the production of glycolytic intermediates to meet the biosynthesis needs of the cells. These intermediates are integrated into various metabolic pathways to generate *de novo* nucleotides, lipids, amino acids, and NADPH [22–24]. For example, the accumulation of glycolytic intermediates promotes the pentose phosphate pathway (PPP), resulting in the generation of NADPH and ribose-5-phosphate. NADPH acts as a reducing agent for lipid, nucleotide, and amino acid biosynthesis. Ribose-5-phosphate is essential for the biosynthesis of nucleic acids. Dihydroxyacetone phosphate resulting from glycolysis is converted to glycerol-3-phosphate, which is crucial for the biosynthesis of the phospholipids and triacylglycerols required for generation of cell membranes. Finally, the NADPH is also used for the reduction of cellular glutathione (GSH) pools and enables the cancer cells to maintain adequate levels of reduced forms of GSH, which is critical for cancer cells resistance against chemotherapeutic agents.

Proliferating cancer cells adapt several molecular mechanisms to maintain high glycolytic flux [23]. First, they upregulate the expression of phosphofructokinase-2 (PFK2), which produces fructose-2,6-bisphospate as a potent allosteric activator of PFK1 to overcome negative allosteric feedback inhibition of high ATP levels on PFK1, a critical driver of glycolytic flux. Second, generation of NAD^+^ from NADH *via* upregulation of LDH is necessary for maintaining glycolytic flux. Finally, cancer cells express higher levels of pyruvate kinase M2 (PKM2) which can be allosterically and covalently inhibited. PKM2 inhibition facilitates glycolytic intermediates upstream of pyruvate into biosynthetic pathways, and phosphoenolpyruvate is subsequently converted to pyruvate through alternative pathways to generate lactate and NAD^+^ [25].

It must point out that aerobic glycolysis is not a specific marker of tumors. It has been well know that fast growing tissues or cells also depend more on glycolysis than on OXPHOS for energy production. The metabolism of stem cells, including embryonic stem cells, hematopoietic stem cells, and induced pluripotent stem cells, shows the Warburg effect [26–31]. Lymphocytes exhibit a striking metabolic shift upon activation. Glycolysis is significantly upregulated during lymphocyte activation, even in the presence of oxygen [3, 32, 33]. Similar to tumors, the increase in glycolysis generates ATP at a faster rate than OXPHOS and supplies metabolic precursors to meet the metabolic requirements of cell proliferation, while producing less reactive oxygen species (ROS) [3, 34]. However, this metabolic shift may not only be necessary for proliferation or survival, but also specifically required for effector function in T cells [35, 36].

## REGULATION OF GLYCOLYSIS IN TUMORS

**Figure 2:**
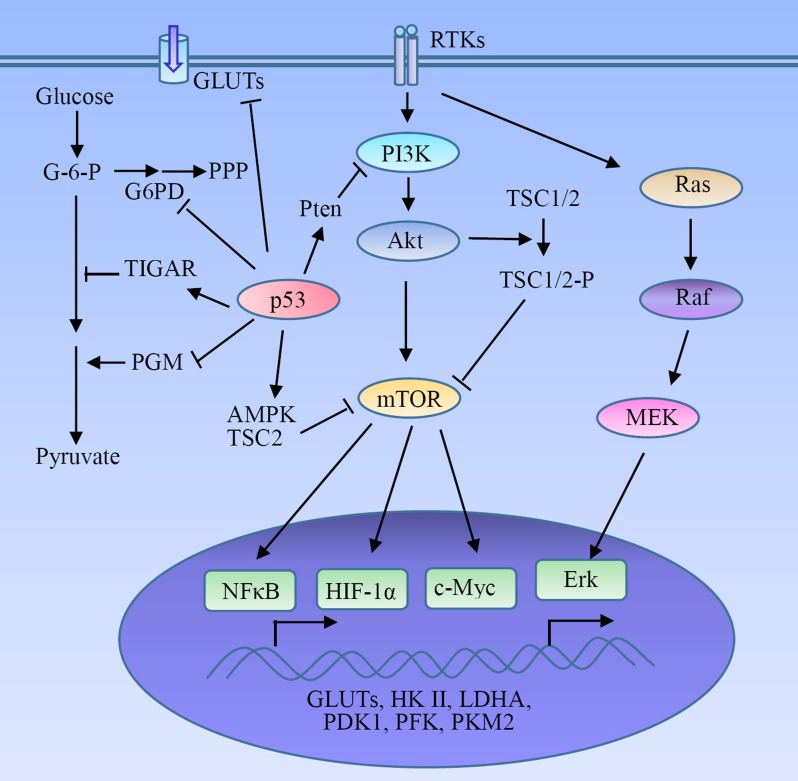
Regulatory mechanism of glycolysis in tumors HIF-1α serves as a key activator of glycolysis through the induction of GLUTs and many glycolytic enzymes. The receptor tyrosine kinases (RTKs)-mediated PI3K-Akt-mTOR signaling pathway plays a pivotal role in the metabolic switch to aerobic glycolysis in tumor cells *via* the activation of HIF-1α, NFκB and c-Myc, and the subsequent expression of glycolytic enzymes. Some oncogenes and tumor suppressors such as Ras and p53 are involved in the regulation of the Warburg effect.

The Warburg effect is a hallmark of cancer. However, the highly glycolytic mechanism of cancer cells remains less clear. Although hypoxia is a main reason resulting in abnormal glycolytic flux in cancer cells, the glycolysis enhances in many tumors under normoxic conditions. The other factors such as oncogenes and related signaling pathways are involved in glycolytic switch in tumors (Table 1). Oncogenes can directly activate hypoxia-inducible factor-1 (HIF-1) and other components of glucose metabolism independently of hypoxia in many cancers (Figure 2).

**Table 1:** Major regulators of aerobic glycolysis in tumor

Key modulators	Via	References
Abhd5	AMPK, GLUT1, HK I, HK II, LDHA, PKM1, p53	[37]
Caveolin 1	Akt-mTOR, GLUT3	[38–40]
CD147	MCT1, MCT4, p53	[41–43]
Ecdysoneless	GLUT4	[44]
FAK	LDH, MCT, PKM2	[45]
GRIM-19	HIF-1α, p53, STAT3, HK II, PDK1, PFK1, PKM2	[46]
GRP78	HIF-1α, PKM2	[47]
HIF-1α	GLUT1, HK II, PDK1, PKM2	[48–52]
HSP40	PKM2	[53]
KLF4	LDHA	[54]
K-Ras	GLUT1	[55]
KSHV	PKM2	[56]
LMP1	LDHA	[57]
miRNAs	GLUT1, GLUT3, HK II, LDHA, PFK, PFKFB3	[58–69]
p53	AMPK, GLUT1, GLUT3, GLUT4, G6PD, PGM, Pten, TIGAR, TSC2	[70–77]
P2×7	GLUT1, HIF-1α, PKM2	[78]
PI3K-Akt-mTOR	c-Myc, HIF-1α, NFκB GLUT1, GLUT3, HK II, LDHA, PFK, PKM2	[48, 79–83]
Skp2	Akt, GLUT1	[84]
Survivin	DNM1L	[85]
TRAP1	c-Src	[86]
Wnt	PDK1	[87]
ZBTB7A	GLUT3, PFKP, PKM	[88]

### HIF-1α

Hypoxia-inducible factor-1α (HIF-1α) contributes greatly to the enhanced glycolysis in tumors. HIF-1α stimulates glycolysis through direct transactivation of glucose transporters (GLUTs) such as GLUT1 and many glycolytic enzymes such as hexokinase II (HK II), pyruvate dehydrogenase kinase 1 (PDK1) and PKM2 [48, 49]. The high glycolytic rate in hypoxic solid tumor is due in part to the greatly increased HIF-1-mediated expression of HK II [50]. Pyruvate dehydrogenase converts pyruvate into acetyl-CoA for tricarboxylic acid (TCA) cycle. PDK1 phosphorylates and inhibits pyruvate dehydrogenase, and subsequently prevents the entry of pyruvate into the TCA cycle [51, 52]. Cancer cells express higher levels of PKM2 over the more catalytically active PKM1, leading to accumulation of cellular carbohydrate metabolites that can be used for the biosynthesis of macromolecules to support the proliferation and rapid growth of tumor cells.

### PI3K-Akt-mTOR pathway

Akt, a serine/threonine kinase promoting cancer growth, activates aerobic glycolysis and renders cancer cells dependent on glycolysis for survival [89]. Regulation of Akt on glycolysis involves HK II [90, 91], PFK2 [92], and GLUT1 [91, 93]. Akt signaling induces HK II expression [90, 91], and phosphorylates and activates PFK2 [92]. PFK2 catalyzes the production of fructose-2,6-bisphosphate, which acts as an allosteric activator of PFK1. Activation of the Akt upregulates GLUT1 gene transcription [91, 93]. HK II and PFK1 are rate-controlling enzymes of glycolysis. GLUT1 is the most widely expressed glucose transporter which is translocated to cell surface induced by Akt to promote glucose uptake. Akt promotes a glycolytic switch under normoxic conditions in tumor cells and was coined the ‘Warburg kinase’ [94]. Enhanced aerobic glycolysis mediated by Akt leads to acquired radioresistance of tumor cells [95]. Deficiency of Skp2, an E3 ligase, impairs Akt activation as well as GLUT1 expression, glycolysis, and cancer progression [84]. Glycolysis promotion induced by Akt does not affect the rate of OXPHOS, implying that this effect is not an adaptation to hypoxia rather than to meet the increased needs of metabolic intermediates required for rapid proliferation of tumor cells. Thus the Akt-mediated aerobic glycolysis is critical for growth and survival of tumor cells. Tumor cells bearing an activated form of Akt undergo rapid cell death when shifted to low-glucose conditions [89]. Akt also phosphorylates and inactivates tumor suppressor TSC2, a negative regulator of mammalian target of rapamycin (mTOR), to promote glycolysis (see discussion below) [96].

PI3K-Akt-mTOR is a major signaling pathway involved in cancer development and progression, which is pivotal in the regulation of aerobic glycolysis and tumor growth. A number of receptor tyrosine kinases (RTKs), such as the epidermal growth factor receptor (EGFR), insulin-like growth factor receptor, and platelet-derived growth factor receptor (PDGFR), can activate PI3K at the cell membrane, initiating the signaling cascade. Upon the PI3K activation, Akt is recruited to the cell membrane and activated. mTOR is a serine/threonine kinase downstream of PI3K-Akt and acts through two separate complexes, mTOR complex 1 (mTORC1) and mTORC2, which play a critical role in tumorigenesis and metabolism [97]. RTK-PI3K-Akt-mTOR signaling cascade is a frequently altered pathway in cancer. PDGFR signaling regulates glycolysis in glioma-derived tumor stem-like cells through the activation of Akt [98]. PI3K-Akt-mTOR signaling is indispensable for the EGFR-mediated regulation of aerobic glycolysis in lung cancer cells [99]. Insulin receptor substrate 2-mediated PI3K signaling selectively inhibits glycogen synthase kinase 3β to promotes glucose uptake and aerobic glycolysis [100]. Pten phosphatase is a negative regulator of PI3K, and its deficiency increases HK II mRNA translation in prostate cancer cells through the activation of Akt-mTORC1-4E binding protein 1 axis [101]. Pten inhibits glycolysis through both PI3K/Akt-dependent and -independent pathways [101, 102].

Metabolic switch to aerobic glycolysis in cancer cells involves the mTOR-mediated expression of glycolytic enzymes through the activation of HIF-1α, NFκB, and c-Myc [79–82]. mTOR was identified as a central activator of the Warburg effect by inducing PKM2 under normoxic condition [82]. Upregulation of PKM2 induced by mTOR is critical for aerobic glycolysis and tumor growth [82]. PKM2 is a rate-limiting glycolytic enzyme, and exclusively expressed in embryonic, proliferating, and tumor cells. mTOR stimulates PKM2 expression through HIF-1α-mediated transcription activation and c-Myc-heterogeneous nuclear ribonucleoproteins (hnRNPs)-dependent regulation of PKM2 gene splicing [82]. hnRNPs were identified as splicing repressors for PKM1. Disruption of PKM2 suppresses mTOR-mediated tumorigenesis. Dual suppression of mTOR and glycolysis synergistically blunts the proliferation and tumor development of mTOR hyperactive cells. TSC1/TSC2 complex negatively regulates the expression of GLUT3 [83]. Loss of TSC1/TSC2 upregulates GLUT3 through the activation of the rapamycin-sensitive mTORC1 signaling. mTORC1 upregulates GLUT3 through the activation of NFκB pathway [83]. Depletion of GLUT3 suppresses aerobic glycolysis, inhibits cell proliferation and colony formation, and attenuates the tumorigenic potential of the cells with aberrantly hyper-activated mTORC1 signaling in nude mice. In addition, a SIRT1-mTOR/HIF-1α glycolytic pathway is required for differentiation of myeloid-derived suppressor cells to the M1 phenotype [103]. MUC16, a transmembrane mucin increases glycolysis in pancreatic cancer through the activation of mTOR and c-Myc [104].

### K-Ras

Activation of K-Ras (G12V) causes mitochondrial dysfunction, leading to a metabolic switch from OXPHOS to glycolysis. Pre-induction of K-Ras expression *in vitro* resulting in glycolytic switch enhances the ability of the transformed cells to form tumor *in vivo* [11]. Mutated K-Ras leads to increased expression of the GLUT1, glucose uptake, glycolysis, and lactate production [55]. K-Ras mutated cells showed increased survival in low-glucose culture condition. They are much sensitive to the toxicity of 3-bromopyruvate (3-BP), a hexokinase inhibitor compared with cells lacking K-Ras mutation [55]. Oncogenic K-Ras has been reported to maintain pancreatic tumors through regulation of glycolysis [105]

### p53

p53, a tumor suppressor, induces cell-cycle arrest and cell death after DNA damage, and thus contributes to the maintenance of genomic stability. p53 plays a critical role in promoting mitochondrial OXPHOS and downregulation of glycolysis [106, 107]. First, p53 represses aerobic glycolysis through regulation of glucose transporters and glycolytic enzymes. p53 directly suppresses the expression of GLUT1 and GLUT4 [108], and indirectly downregulates GLUT3 [70]. p53 promotes the ubiquitination-mediated degradation of phosphoglycerate mutase (PGM). Loss of p53 increases PGM level and glycolysis [71]. p53 also negatively regulates glycolysis *via* TIGAR (TP53-induced glycolysis and apoptosis regulator). TIGAR dephosphorylates fructose-2,6-bisphosphate to fructose-6-phosphate and diverts glucose catabolism to the PPP [72, 73]. Second, p53 regulates glucose metabolism through inhibiting glucose-6-phosphate dehydrogenase (G6PD), the first and rate-limiting enzyme in the PPP [74]. Third, p53 regulates glycolysis through inducing a group of target genes to negatively regulate PI3K-AKT-mTOR pathway. For example, p53 induces Pten to inhibit the PI3K-AKT signaling [75]. p53 activates AMPK, a major upstream negative regulator of mTOR, leading to the downregulation of the mTOR activity [75, 76]. p53 also induces TSC2 to negatively regulate the mTOR activity. It has recently been found that p53 induces its target RRAD, which in turn inhibits the GLUT1 translocation to the plasma membrane and represses glycolysis in lung cancer cells [77].

p53 frequently mutates in tumors. Recent studies have revealed that tumor-associated mutant p53 (mutp53) drives the Warburg effect under normoxia [109]. mutp53 stimulates the Warburg effect in cultured cells through promoting GLUT1 translocation to the plasma membrane, which is mediated by activated RhoA and its downstream effector ROCK. Inhibition of RhoA-ROCK-GLUT1 signaling abolishes role of mutp53 in inducing the aerobic glycolysis, and inhibition of glycolysis in tumor cells greatly compromises mutp53-promoting tumorigenesis [109].

### AMPK

Adenosine monophosphate activated protein kinase (AMPK) is a critical cellular energy sensor detecting the balance between ATP production and consumption in eukaryotic cells [110]. Once activated by energetic stress *via* increases in AMP:ATP and ADP:ATP ratios, AMPK changes the metabolic process from anabolic condition to a catabolic state [111, 112]. AMPK activation switches off most anabolic processes, such as synthesis of lipids, carbohydrates, ribosomal RNA, and proteins [111]. AMPK downregulates protein synthesis through the repression of mTOR-dependent mRNA translation [113–115]. This includes downregulation of ribosomal proteins, HIF-1α, and thus expression of the glycolytic enzymes and transporters required for the Warburg effect [116, 117]. AMPK activation therefore promotes the oxidative metabolism typical of quiescent cells, rather than the aerobic glycolysis observed in most proliferating cells including tumor cells [112, 118]. AMPK is a potential target for cancer prevention and treatment as well as anti-inflammatory drug [119, 120].

### c-Myc

c-Myc acts as a transcription factor involved in the control of cell proliferation, differentiation, and apoptosis. By using c-Myc transgenic model, Valera et al. revealed that the expression of glycolysis-related enzymes, such as glucokinase, PFK2, and PK, are increased in the liver of the transgenic mice, suggesting that c-Myc is involved in the control *in vivo* of carbohydrate metabolism [121]. c-Myc promotes glucose uptake and glycolysis through the upregulation of GLUT1 and many glycolytic enzymes, including HK II and PFK and lactate dehydrogenase A (LDHA) [48]. c-Myc directly transactivates expression of GLUT1 [122], MCT1, and MCT2 [123]. c-Myc can also enhance expression of MCT1 *via* transcriptionally repressing miR-29a and miR-29c [123]. hnRNPs proteins, polypyrimidine tract binding protein (PTB), hnRNPA1, and hnRNPA2, bind to PKM pre-mRNA and switch PKM splicing to favor PKM2 variant [124, 125]. c-Myc upregulates transcription of PTB, hnRNPA1, and hnRNPA2, ensuring a high PKM2/PKM1 ratio. LDHA was identified as another c-Myc responsive gene [126, 127]. Knockdown of c-Myc reduces the expression of LDHA, lactate production, and glucose consumption [127]. LDHA overexpression is required for c-Myc-mediated transformation and tumor growth [126, 127].

Human pituitary tumor-transforming gene (PTTG), a proto-oncogene, influences glycolysis through regulation of c-Myc [128]. PTTG knockdown in ovarian cancer cells results in the downregulation of c-Myc and several crucial proteins involved in aerobic glycolysis, including PKM2, LDHA, and GLUT1. Overexpression of c-Myc could prevent the PTTG knockdown-induced metabolic shift [128], suggesting that PTTG regulates the metabolic switch *via* the c-Myc pathway. N-Myc downstream regulated gene 2 (NDRG2), a tumor suppressor gene, significantly suppresses the expression of GLUT1, HK2, PKM2, and LDHA, leading to inhibition of glucose consumption and lactate production in colorectal cancer cells [129]. c-Myc mediates the inhibition of glycolysis by NDRG2. NDRG2 inhibits the expression of c-Myc by suppressing the production of β-catenin, which can transcriptionally activate c-Myc. Therefore NDRG2 functions as an essential regulator in glycolysis *via* repression of c-Myc [129].

### miRNAs

Accumulating evidence suggests that microRNAs (miRNAs) interact with oncogenes/tumor suppressors and induce the aerobic glycolysis in cancer cells. miRNAs can regulate glucose transporters and glycolytic enzymes [130]. miR-144 inhibits GLUT1 expression through targeting its 3′-untranslated region in ovarian cancer cells [64]. miR-22 and miR-1291 also directly target GLUT1 in breast cancer [65] and renal cell carcinoma [66], respectively. miR-195-5p directly targets GLUT3 and suppresses glucose uptake in bladder cancer cells [67]. Several miRNAs regulate glycolysis *via* targeting HK II. miR-143 inhibits the expression of HK II in head and neck squamous cell carcinoma (HNSCC)-derived cells [68] and colon cancer cells [69]. miR-143, downregulated by mTOR activation, regulates cancer glycolysis and inhibits cancer cell proliferation and tumor formation *via* targeting HK II in human lung cancer [58]. miR-143 is downregulated in glioma tissues and glioblastoma stem-like cells (GSLCs). It inhibits glycolysis by directly targeting HK II and depletes GSLCs stemness [59]. miR-155 upregulates HK II *via* repressing miR-143 and activating the signal transducer and activator of transcription 3 (STAT3), a transcriptional activator for HK II [131]. miR-29b negatively regulates Akt expression, causing HK II/PKM2 downregulation and leading to a decreased the Warburg effect and slowed ovarian cancer progression [132]. miR-199a-5p regulates glycolysis and lactate production by targeting HK II [60]. It is downregulated in human liver cancer and is negatively associated with malignancies. The upregulation of HIF-1α under hypoxic conditions suppresses the expression of miR-199a-5p and promotes glycolysis.

LDHA, PFK, and 6-phosphofructo-2-kinase/fructose-2,6-bisphosphatase-3 (PFKFB3) are other targets for miRNAs. LDHA is frequently overexpressed in tumor cells. Knockdown of LDHA results in decreased lactate and ATP production, glucose uptake and cell growth. Several miRNAs target LDHA and regulate glycolysis in cancer cells [61]. miR-320a directly regulates PFK expression and sequential lactate production [63]. miR-26b and miR-206 inhibit tumor growth *via* the downregulation of PFKFB3-driven glycolysis [62, 133]. Overexpression of miR-26b represses PFKFB3 expression and regulates the expression of LDHA, GLUT1, and markers of invasion and cell cycle. miR-21 diminishes aerobic glycolysis in bladder cancer cells *via* the Pten/PI3K/AKT/mTOR axis [134].

### Other regulators

Wnt signaling regulates glycolysis and angiogenesis through PDK1 [87]. Interference with Wnt signaling in colon cancer cells reduces glycolytic metabolism and suppresses tumor growth. PDK1 overexpression in Wnt-inhibited cancer cells rescues glycolysis and vessel growth. Heat shock proteins (HSPs) including HSP40 [53] and TNF receptor-associated protein 1 (TRAP1) [86], a member of the HSP90 chaperone family, are involved in the regulation of metabolic switch between OXPHOS and aerobic glycolysis in tumors. Focal adhesion kinase (FAK), a key transmitter of growth factor and anchorage stimulation, enhances glycolysis and decreases mitochondrial respiration through the upregulation of glycolytic proteins enolase, PKM2, LDH and MCT [45]. Attenuation of FAK-enhanced glycolysis decreases cell viability and reduces growth of tumor xenografts. HSulf-1, a putative tumor suppressor, is a negative regulator of glycolysis. Silencing of HSulf-1 expression in ovarian cancer cells increases glucose uptake and lactate production by upregulating GLUT1 and glycolytic enzymes HK II and LDHA [135]. The P2X7 receptor (P2X7R), an ATP-gated cation channel, is a key modulator of aerobic glycolysis [78]. Forced expression of P2X7R in HEK293 cells increases phosphorylated Akt and HIF-1α expression; upregulates GLUT1, GAPDH, PFK, PKM2, and PDK1; and inhibits pyruvate dehydrogenase activity.

CD147 is a transmembrane glycoprotein and plays an important role in tumorigenicity, invasion, and metastasis. CD147 promotes glycolysis and tumor progression in epithelial solid tumors through the regulation of the p53-dependent signaling pathway and MCT [41–43]. Plasma membrane-associated protein Caveolin 1 enhances aerobic glycolysis *via* regulation of Akt-mTORC1-GLUT3 signaling [38–40]. Glucose regulated protein 78 (GRP78) is involved in the modulation of tumor aerobic glycolysis [47]. Overexpression of GRP78 induces inactivation of NF-κB pathway, and subsequently alters the expression of PKM2 and HIF-1α. α/β-hydrolase domain-containing 5 (Abhd5) is an intracellular lipolytic activator. Suppressing Abhd5-mediated intracellular lipolysis stimulates aerobic glycolysis in cancer cells [37]. Decreased expression of the gene associated with retinoid-interferon induced mortality-19 (GRIM-19) promotes aerobic glycolysis and cell proliferation in HNSCC [46]

ZBTB7A, a member of the POK (POZ/BTB and Krüppel) transcription repressor family, acts as a novel tumor suppressor by directly suppressing glycolysis [88]. ZBTB7A-deficient tumors progress very fast and are extremely sensitive to glycolysis inhibition. A novel Krüppel-like factor 4 (KLF4) regulates aerobic glycolysis in pancreatic cancer through negatively regulating the transcription of LDHA [54]. KLF4 overexpression significantly attenuates the aerobic glycolysis and cancer cell growth. Antiapoptotic protein Survivin induces a switch of OXPHOS to aerobic glycolysis in tumor cells and may acts as a target for glycolysis inhibition [85, 136]. Ecdysoneless regulates aerobic glycolysis *via* GLUT4 in pancreatic cancer cells [44].

Some viruses and their encoded proteins even regulate glucose metabolism in cancer cells. Kaposi's sarcoma (KS) is a vascular neoplasm caused by infection of Kaposi's sarcoma-associated herpesvirus (KSHV). KSHV induces aerobic glycolysis through HIF-1-dependent upregulation of PKM2 in Kaposi's sarcoma [56]. The EBV-encoded latent membrane protein 1 (LMP1) increases cellular uptake of glucose, enhances LDHA activity and lactate production, contributing to aerobic glycolysis [57].

## POTENTIAL TARGETS FOR TUMOR THERAPY

Although the mechanism triggering the Warburg effect in tumors is not well clear, the oncogenic stress including activation of oncogenes and inactivation of tumor suppressors plays a critical role. Aerobic glycolysis contributes to the diverse aspects of tumor development, progression and prognosis [89, 137, 138]. The reversing the Warburg effect greatly compromises the tumorigenicity of tumor cells [139, 140], suggesting that targeting the metabolic changes could be an effective strategy for cancer treatment. Actually, many attempts have been made and some molecular targets have shown their potential in the cancer therapy. Several small molecules, as a single agent or in combination with other therapeutic modalities, exhibit promising anticancer activity both *in vitro* and *in vivo*. Of them, lonidamine, 2-deoxyglucose (2-DG), dichloroacetate, and 3-BP have been clinically tested (Table 2). Malignant cells exhibited the Warburg effect become dependent on *de novo* lipogenesis, which sustains rapid proliferation and resistance to cellular stress. Anti-tumor activity of some small molecules selectively targets the Warburg effect and lipogenesis [141, 142].

**Table 2:** The potential targets and the corresponding chemicals

Targets	Potential chemicals	Development stage	Cancer types	References
GLUTs	Fasentin, phloretin, WZB117	Animal tested	Liver, lung	[143–145]
HK II	2-DG	Clinical trial terminated	Lung, osteosarcoma, prostrate	[146, 147]
	3-BP	Clinical trial phase I	Liver, stomach	[148, 149]
	Lonidamine	Phase III completed	Breast, glioblastoma, lung, prostrate	[150]
	FV-429	Experimental drugs	Breast	[151]
	Clotrimazole	Experimental drugs	Breast	[152]
PFK	3PO	Experimental drugs	Bone marrow, epithelium, lung	[153, 154]
GAPDH	3-BP	Experimental drugs	Liver	[155–157]
PK	Shikonin, siRNA	Experimental drugs	Multiple cancers	[158–161]
LDHA	FX11	Experimental drugs	Lymphoma, pancreas	[162]
	Oxamate	Experimental drugs	Breast	[163]
	N-hydroxyindole derivates	Experimental drugs	Multiple cancers	[164]
MCT1	α-cyano-4-hydroxy-cinnamic acid	Experimental drugs	Glioma cells	[165]
PDK	Dichloroacetate	Clinical trial phase I	Multiple cancers	[166, 167]

### Glucose transporters (GLUTs)

GLUTs transport glucose into the cancer cells and are considered as putative targets. GLUT1 is upexpressed in many cancer types. The several small molecules are identified to inhibit GLUT1 and kill tumor cells in preclinical models [145, 168]. As GLUTs are ubiquitously expressed proteins, blockade of GLUTs will inevitably disrupt glucose consumption in normal tissues. It would be challenging to inhibit a specific isoform associated with tumor cells within an acceptable therapeutic window. In addition, GLUTs have been explored as receptors to import drug-loaded nanoparticles across the blood-brain barrier [169].

### Hexokinase II (HK II)

HKs catalyze the first step of glycolysis, a rate-limiting step. There are four mammalian isoforms (I to IV) that are usually expressed at low levels in cells [170]. Of them, HKs, HK II is specially expressed in insulin-sensitive tissues such as muscle and adipose. It has a high affinity (low Km) for glucose facilitating the glycolysis in low serum glucose level. HK II is a key mediator of aerobic glycolysis and promotes tumor growth [171]. HKII is overexpressed in many tumor cells. Systemic targeting of HK II blocks tumor growth without adverse physiologic consequences [172]. These findings suggest that HK II is another potentially attractive therapeutic target for cancer.

There are different strategies to dysfunction HK II. Glucose analog 2-DG shows promising therapy effects in combination treatments with other anticancer agent [146, 147]. Intracellular accumulation of glucose analogs could inhibit HK *via* feedback inhibition mechanism. Inhibiting glycolysis with 2-DG reduces tumor cell radioresistance [95]. Some small molecules such as 3-BP and lonidamine are direct inhibitors of HK enzyme. Preclinical studies demonstrate that 3-BP inhibits HK II in human cancer and is a promising anticancer drug targeting glycolysis [148, 149]. Lonidamine has completed phase III clinical trials in breast and lung cancers, and a trend for higher tumor responses and better survival parameters was observed when combined with chemotherapy [150, 173]. FV-429, a newly synthesized flavonoid, inhibits glycolysis in human breast cancer cells through inhibiting Akt-mediated phosphorylation of HK II and downregulation of its activity [151]. Clotrimazole, an azole derivative with promising anti-cancer effects, decreases glucose uptake and inhibits the major glycolytic enzymes, HK, PFK1, and PK in human breast cancer cells [152].

### Phosphofructokinase (PFK)

PFK1, another rate-limiting enzyme of glycolysis, is activated by fructose-2,6-bisphosphate. The latter is regulated by the activity of a family of bi-functional enzymes, 6-phosphofructo-2-kinase/fructose-2,6-bisphosphatase isozymes (PFKFB1-4). PFKFB3 isozyme is constitutively expressed by tumor cells and required for the high glycolytic rate. A small molecule inhibitor of PFKFB3, 3-(3-pyridinyl)-1-(4-pyridinyl)-2-propen-1-one (3PO), suppresses glycolytic flux and is cytostatic to tumor cells [153]. A combination of 3-PO with ascorbic acid has been shown synergistic activity in non-small cell lung cancer cells [154].

### Glyceraldehyde 3-phosphate dehydrogenase (GAPDH)

In addition to being a glycolytic enzyme, GAPDH has multiple functions such as posttranscriptional control of T cell effector function [35]. GAPDH catalyzes the first step of glycolysis pay-off phase and is an alternative promising therapeutic target. NADH produced during this step plays a critical role in the cellular redox balance. Several GAPDH inhibitors have been tested for their efficacy, and the pyruvate analog 3-BP has been demonstrated to be the most promising one [155]. 3-BP inhibits tumor glycolysis and dramatically reduces intracellular ATP level, with excellent specificity and selectivity for GAPDH [156, 157].

### Pyruvate kinase (PK)

Muscle type of pyruvate kinase (PKM) is one of the key mediators of the Warburg effect and tumor metabolism. PKM1 and PKM2 are two major isoforms, which are alternative splice products of the PKM gene. PKM2 is upregulated in many tumors [139, 174]. High expression of PKM2 correlates with shorter recurrence-free survival in pancreatic ductal adenocarcinoma patients. Switching PKM splicing to favor PKM1 variant in drug resistant pancreatic cancer cells rescues sensitivity to gemcitabine and cisplatin, suggesting that PKM2 expression is required to withstand drug-induced genotoxic stress [175]. Some small molecule inhibitors including siRNA specific for PKM2 have been tested, and the preclinical studies demonstrate that PKM2 could be a potential therapeutic target [158–161]. Specific knockdown of the PKM2 results in decreased viability and increased apoptosis in multiple cancer cells and causes substantial tumor regression of established xenografts [159]. However, Inhibiting PKM2 could allow glycolytic intermediates to accumulate and feed biosynthetic pathways, resulting in tumor promotion [23]. PKM2 is regulated by cellular oxidative stress [176]. Increase of intracellular ROS concentration causes inhibition of the PKM2 *via* oxidation of Cys358, promoting diversion of glycolytic intermediates into the PPP to generate sufficient reducing potential for detoxification of ROS. Oxidation-resistant PKM2 mutant exhibits increased sensitivity to oxidative stress and impaired tumor formation in a xenograft model [176]. The small molecule PKM2 activators may also be used to interfere with cancer cell metabolism for therapeutic purposes.

It was previously thought that PKM1 is specific for non-proliferating tissues and PKM2 for proliferating tissues, and that the isoform switch from PKM1 to PKM2 results in high PKM2 expression in tumors, providing a great advantage to tumor cells [139]. However, this traditional view is challenged by recent studies. It has demonstrated that PKM2 is not specific for tumors [177]. The isoform switch does not occur during tumorigenesis [177] or is tissue specific, only occurred in glioblastomas and not in other tumor types [178]. PKM1 even switches to other isoforms rather than PKM2. Instead of the isoform switch, the upregulation of PKM2 is probably due to elevated transcriptional levels of the entire PKM gene [179]. PKM2 could be non-essential for *in vivo* tumor growth and maintenance [180].

### Lactate dehydrogenase (LDH) and monocarboxylate transporters (MCTs)

LDH is a tetramer composed of two different subunits, LDHA and LDHB. LDH catalyzes the final glycolytic step that converts pyruvate into lactate. The accumulation of lactate and subsequent low intracellular pH is extremely harmful. Inhibition of LDH has demonstrated promising effects in preclinical studies. LDHA inhibitor FX11 lowers intracellular ATP levels, which significantly induces oxidative stress and inhibits tumor progression [162]. Another LDHA inhibitor, oxamate, re-sensitizes taxol-resistant cancer [163]. Some N-hydroxyindole-based LDHA inhibitors show an effective anti-proliferative activity in a series of cancer cells [164]. The lactate is exported into the extracellular space *via* specific transporters monocarboxylate transporters (MCTs). Blocking lactate export by disrupting MCT1 function leads to an accumulation of intracellular lactate that rapidly disables tumor cell growth and glycolysis [181]. A small molecule inhibitor of lactate transport, α-cyano-4-hydroxy-cinnamic acid, has been shown to inhibit tumor invasiveness and induce tumor necrosis [165].

The significance of LDHB in tumor development is elusive. Expression of LDHB is suppressed in many kinds of cancer due to promoter hypermethylation [182]. LDHB acts as a suppressor of glycolysis and suppresses pancreatic cancer progression [183]. Decreased expression of LDHB in liver cancer enhances cell invasiveness *via* mitochondrial defects [184]. In contrast, LDHB is required for the growth of K-Ras-dependent lung adenocarcinomas [185] and hyperactive mTOR-mediated tumorigenesis [186]. The tumor suppressor drs regulates glucose metabolism *via* LDHB [187]. Downregulation of drs may contribute to the enhanced glycolysis *via* increased LDHB expression, which is closely associated with malignant progression of cancer cells.

### miRNAs

Several miRNAs are engaged in glycolysis, providing a potential therapeutic strategy. For example, miR-143 targets HK II to regulate glucose metabolism in multiple cancer cells. miR-143 inhibits proliferation of GSLCs under hypoxic conditions and decreases tumor formation capacity of GSLCs *in vivo*. miR-143 overexpression promotes differentiation of GSLCs. A combination of miR-143 and glycolysis inhibitor 2-DG has synergistic effects against GSLCs, suggesting that miR-143 is a potential therapeutic target for glioblastoma treatment [59]. miR-199a-5p directly targets the 3′-untranslated region of HK II, indicating that it acts as a suppressor for glucose metabolism [60].

## CONCLUSIONS

During last 1-2 decades a great progress has made toward understanding the mechanism why tumors have a greater reliance on glycolysis for energy than normal tissues. Some oncogenes and corresponding pathways have been proven to be involved in metabolic switch in tumors. This makes tumor cell glycolysis pathway an attractive target for tumor therapy. Several promising preliminary studies illustrate that small molecule inhibitors targeting glycolytic enzymes exert an effective antitumor activity. However, there is a long way to go to uncover the mystery of the Warburg effect. First, we have to evaluate the exact contribution of individual regulator on the glycolytic shift in tumor. It is therefore of great interest to figure out potential master regulator or key driver from a large amount of participators. Second, in terms of cancer therapy or drug development, the specificity of agents mentioned above remains a critical challenge because many glycolytic enzymes are expressed in normal tissues. It remains to verify whether targeting these enzymes will exhibit an effective antitumor effect without significant toxicity to normal tissues. Although several glycolytic inhibitors are in preclinical and clinical development, but none has reached an approved status. Third, it is believed that a small portion of highly tumorigenic cancer cells with stem-like properties is resistant to chemotherapy and may be responsible for the recurrence of cancer after treatment. These cancer cells highly depend on glycolysis for ATP generation and prefer for hypoxia to maintain their stemness and tumor forming capacity. Combination of standard chemotherapeutic agents with glycolytic inhibition is effective in killing the tumor-initiating cells and inhibits tumor formation *in vitro* and *in vivo*. Inhibition of glycolysis is therefore an effective strategy to remove residual cancer stem cells and overcome drug resistance [188, 189].

## Abbreviations

Abhd5: α/β-hydrolase domain-containing 5
Aldo: aldolase
AMPK: adenosine monophosphate activated protein kinase
3-BP: 3-bromopyruvate
2-DG: 2-deoxyglucose
EGFR: epidermal growth factor receptor
Eno: enolase
FAK: focal adhesion kinase
GAPDH: glyceraldehyde 3-phosphate dehydrogenase
GLUTs: glucose transporters
G6PD: glucose-6-phosphate dehydrogenase
GRIM-19: gene associated with retinoid-interferon induced mortality-19
GRP78: glucose regulated protein 78
GSH: glutathione
GSLCs: glioblastoma stem-like cells
HIF-1α: hypoxia-inducible factor-1α
HK: hexokinase
HK II: hexokinase II
hnRNPs: heterogeneous nuclear ribonucleoproteins
HNSCC: head and neck squamous cell carcinoma
HSPs: heat shock proteins
KLF4: Krüppel-like factor 4
KSHV: Kaposi's sarcoma-associated herpesvirus
LDH: lactate dehydrogenase
LDHA: lactate dehydrogenase A
LMP1: latent membrane protein 1
MCT: monocarboxylate transporter
miRNAs: microRNAs
mTOR: mammalian target of rapamycin
mTORC1: mTOR complex 1
NADH: nicotinamide adenine dinucleotide
NDRG2: N-Myc downstream regulated gene 2
OXPHOS: oxidative phosphorylation
PDGFR: platelet-derived growth factor receptor
PDK1: pyruvate dehydrogenase kinase 1
PFK: phosphofructokinase
PFKFB3: 6-phosphofructo-2-kinase/fructose-2,6-bisphosphatase-3
PGI: phosphoglucose isomerase
PGK: phosphoglycerate kinase
PGM: phosphoglycerate mutase
PK: pyruvate kinase
PKM: muscle type of pyruvate kinase
PKM2: pyruvate kinase M2
3PO: 3-(3-pyridinyl)-1-(4-pyridinyl)-2-propen-1-one
PPP: pentose phosphate pathway
PTB: polypyrimidine tract binding protein
PTTG: human pituitary tumor-transforming gene
P2X7R: P2X7 receptor
ROS: reactive oxygen species
RTKs: receptor tyrosine kinases
STAT3: signal transducer and activator of transcription 3
TCA: tricarboxylic acid cycle
TIGAR: TP53-induced glycolysis and apoptosis regulator
TPI: triose phosphate isomerase
TRAP1: TNF receptor-associated protein 1.
